# Angiolipoma in the Head and Neck: Imaging, Diagnosis and Management

**DOI:** 10.3390/medicina56060283

**Published:** 2020-06-10

**Authors:** Vadim Reiser, Bahaa Haj Yahya, Gavriel Chaushu, Ilana Kaplan, Yafit Hamzani

**Affiliations:** 1 Department of Oral and Maxillofacial Surgery, Rabin Medical Center—Beilinson Hospital, Petach Tikva 49100, Israel; vadik.raiser@gmail.com (V.R.); bahaa.hag@gmail.com (B.H.Y.); gabi.chaushu@gmail.com (G.C.); 2 Department of Oral and Maxillofacial Surgery, The Maurice and Gabriela Goldschleger School of Dental Medicine, Tel Aviv University, Tel Aviv 39040, Israel; 3 Department of Oral Pathology, Rabin Medical Center—Beilinson Hospital, Petach Tikva 49100, Israel; Dr.ilanakaplan@gmail.com

**Keywords:** lipoma, angiolipoma, ultrasound, biopsy, neck

## Abstract

Angiolipoma, distinguishable from other lipomas by its excessive degree of vascular vessels, are rare in the head and neck and require unique management. A slow growing mass, located underneath the inferior border of the right mandibular angle of a 51-year-old female, was excised under general anesthesia. Unexpected excessive bleeding during the excision was observed and the histological specimen was diagnosed as angiolipoma. As shown in this case report, pre-operative imaging modalities have a crucial influence and are sufficient to diagnose and manage angiolipomas. The “Gold standard” treatment is excision with clear margins and bleeding management should be taken into account according to appropriate differential diagnosis.

## 1. Introduction

Lipomas are the most common benign mesenchymal tumors, composed of mature adipocytes. They are defined as a well-defined mass, surrounded by thin fibrous capsule, usually asymptomatic, slow-growing and soft [1]. Lipomas can be sub-classified into different histological subtypes based on the appearance of the associated stroma. Angiolipoma, spindle cell lipoma, myelolipoma, chondrolipoma, and myxolipoma are some of the histological variants of lipoma reported in the literature [2]. Around 13% of all lipomas occur in the head and neck, including cheek, tongue, palate, parotid gland, neck, and larynx [3,4,5,6,7,8,9,10].

Unlike the majority of lipomas, which are encapsulated, angiolipomas may be either encapsulated or non-encapsulated. Moreover, they can be differ from other lipomas by the excessive degree of vascular proliferation mixed with mature adipocytes, as well as their ability to infiltrate the surrounding tissues.

The present article reports clinical, radiological, and histological features of an angiolipoma involving posterolateral aspect of the neck of a 51 years old female.

## 2. Case Presentation

In May 2019, a healthy 51-year-old female was referred to the Rabin Medical Centre for the evaluation of a tender mass located 2 cm underneath the inferior border of the right mandibular area, in the posterolateral aspect of the right neck. The patient denied tobacco, alcohol, and illicit drug use. Thirteen years previously, she noticed a small mass in the same area that had progressively slowly increased in size and presented an aesthetic disturbance. Over the weeks prior to her referral, the mass had become tenderer to palpation and raised concern. Clinical examination revealed well-circumscribed, firm and mobile mass, of 4 cm diameter, located in the posterolateral aspect of the right neck. The mass was separated from the surrounding tissues and the overlying skin. There was no audible bruit or liquid flow that suggested a vascular nature. Facial nerve function and cervical lymph nodes were examined and found to be normal. Intra-oral examination revealed normal salivary flow from the orifices of the glands and normal soft tissue appearance. The preoperative clinical findings suggested a benign tumor, favoring lipoma.

Neck ultrasound (US) examination, conducted in August 2006 and May 2007, revealed a well-defined solid hypo-echogenic subcutaneous mass located in the right mandibular angle, 0.93 × 2.52 cm in size. Fine-needle aspiration (FNA), performed in March 2007, exposed blood cells but did not yield diagnostic material or a definitive diagnosis. Following nine additional years without adequate medical follow up, the patient was referred to additional neck US in March 2016, which revealed increase in lesion size to 1.45 × 4 cm, with 1.85 cm depth. Moreover, the mass was defined by the radiologist as well defined; heterogenic; cystic and hypo echogenic which evolve blood flow inside and around the mass.

**Figure 1:**
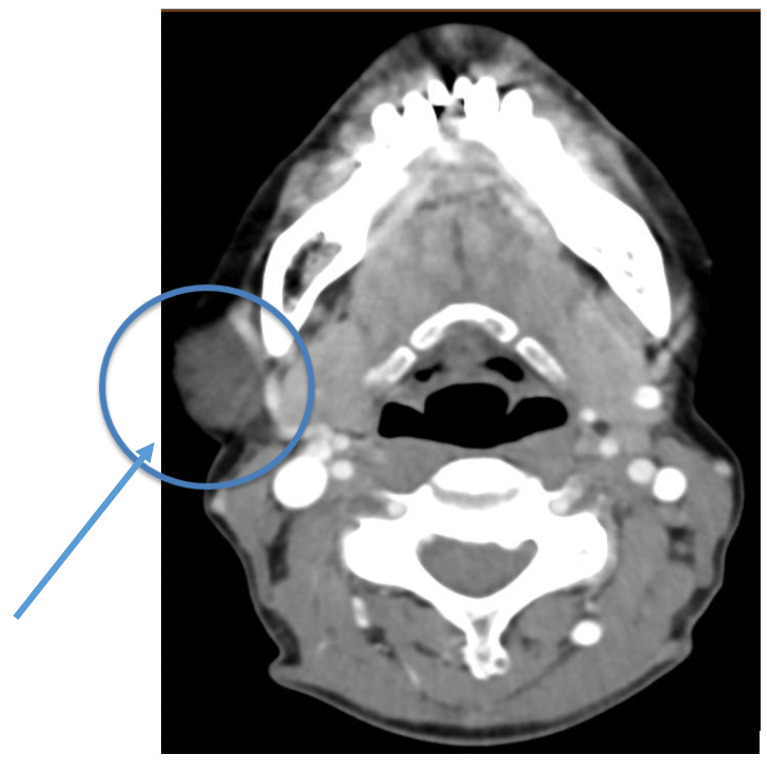
Contrast-enhanced axial section of pre-operative cervical Computed Tomography (CT) scan, demonstrating a well circumscribed middle attenuation mass with peripheral blood vessels enhancement, located in proximity to the superficial parotid lobe (round blue line and arrowhead).

Pre-operative contrast-enhanced Computed Tomography (CT) scanning performed around the referral time, revealed a 4 cm diameter, well-demarcated, middle-intensity (between liquid and soft tissue) homogeneous mass adjacent to the parotid gland and the posterior edge of masseter muscle (Figure 1). The radiologist’s impression was that the lesion is a-vascular but has small blood vessels around it and mentioned that tooth artifacts challenged the examination diagnosis process. Initial differential diagnosis was parotid gland tumor and lipoma.

**Figure 2:**
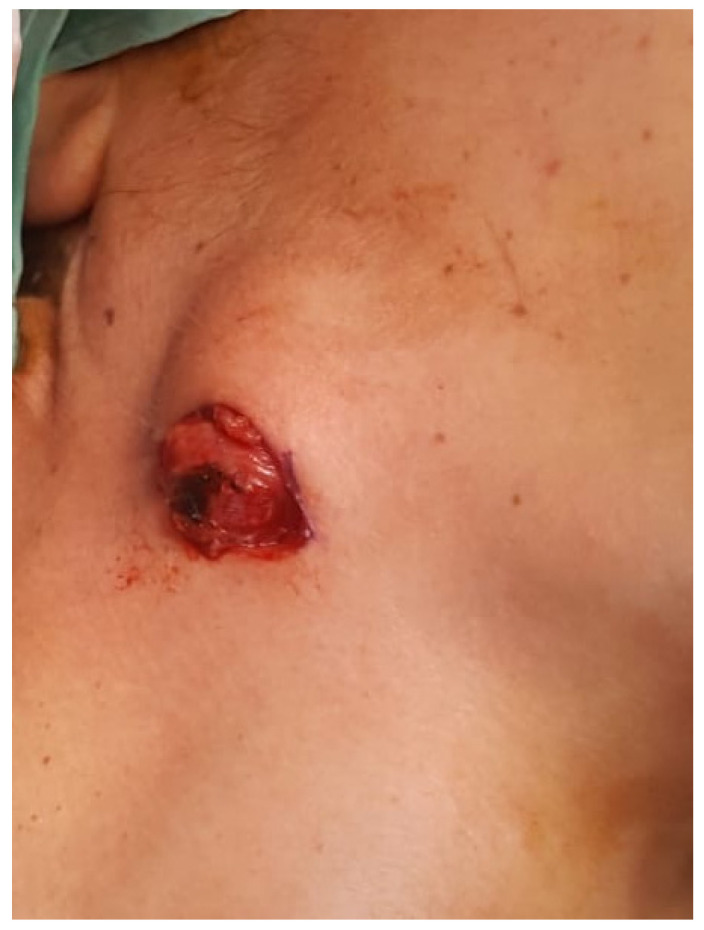
Angiolipoma of the right neck, located underneath the inferior border of the mandible.

**Figure 3:**
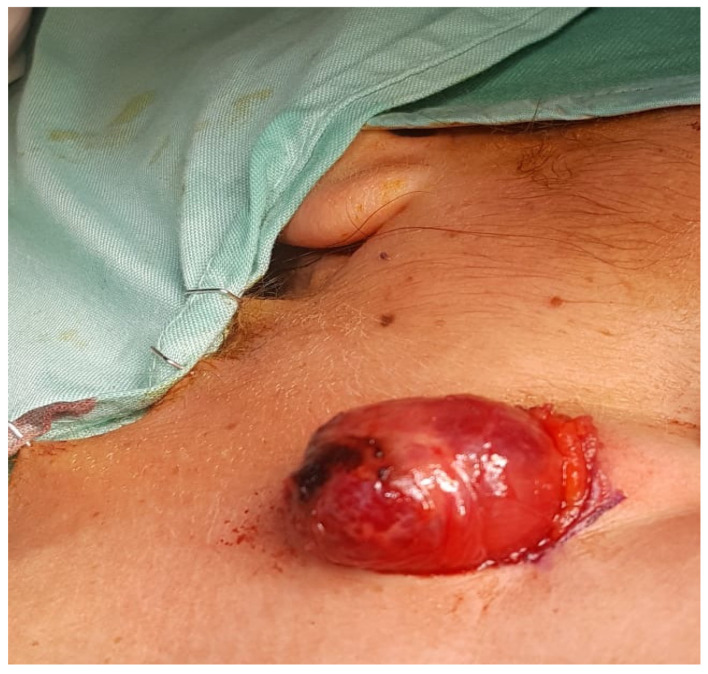
Exposed non-encapsulated, reddish and lobulated mass.

Under general anesthesia surgery, performed 3 months following her referral, the mass was excised, performing a sub-platysma dissection and managing excessive bleeding during the surgery. The mass was located underneath the posterior edge of the masseter muscle, in contact with the right posterior digastric muscle and with the superficial parotid lobe. Clinical findings revealed non-encapsulated, reddish and lobulated mass (Figure 2 and Figure 3). Post-operative recovery was fair, with normal facial nerve function and no evidence of recurrence during 5 months follow up.

**Figure 4:**
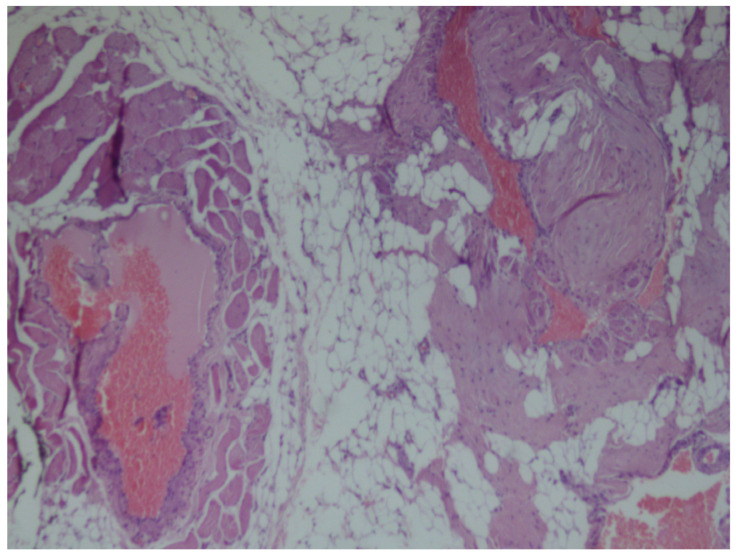
Histopathological micrograph exhibiting a non-encapsulated mass composed of various sized irregular vascular channels surrounded by mature adipose tissue (Hematoxillin and Eosin, original magnification ×40).

**Figure 5:**
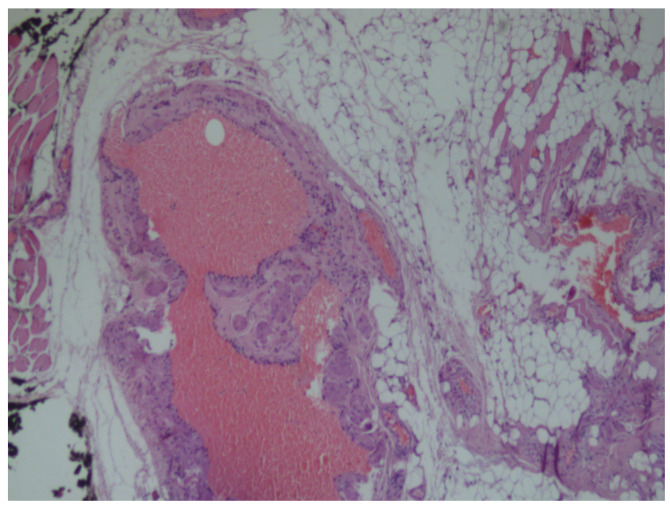
At a higher magnification, the irregular tortuous architecture of the vascular component is evident (Hematoxillin and Eosin, original magnification ×100).

Histopathologic examination of non-encapsulated, most probably infiltrating intramuscular angiolipoma. Microscopically, the tumor was composed of convolutes thin-walled blood vessels, with clusters of mature adipocytes, and a prominent perivascular arrangement of the adipose tissue (Figure 4 and Figure 5).

## 3. Discussion

Lipomas, the most common mesenchymal tumors of soft tissue, are uncommon in the head and neck region [11]. Representing 1–5% of all neoplasms of the oral cavity, lipomas usually present as painless, soft, round, and mobile masses [2]. Only 16% of lipomas appear in the oral and maxillofacial area. A prominent component of proliferating blood vessels is suitable for angiolipoma diagnosis.

Angiolipoma are benign mesenchymal tumors composed of mature lipocytes and vessels representing 5% to 17% of lipomas [12,13]. Angiolipomas were first classified in 1974 by Lin and Lin into noninfiltrating and less-frequent infiltrating [14,15,16]. The former lesions are encapsulated and lack evidence of invasion into surrounding tissues. These lesions are well circumscribed, generally less than 4 cm in size, and appear more frequently in adolescents and young adults [14]. Infiltrating angiolipomas lack a capsule and are more common in elderly patients. They are characterized by the invasion of adjacent structures and the difficulty in separating the masses from the surrounding tissue [17]. Although considered benign, inadequate excision of infiltrating angiolipoma can lead to recurrence [14]. A search of the literature, between 1980 and 2019, found only 42 cases of angiolipoma of the head and neck, seven of which originated in the neck [1,2,9,12,18].

Physical examinations of the neck that reveal soft, mobile, tender, and slow growing masses, should be considered by the physician under the following differential diagnosis: hemangioma; lipoma; Kaposi’s sarcoma; angiosarcoma; leiomyoma; neurilemma [19,20]. Various diagnostic modalities can be used—US, contrast-enhanced CT scan and magnetic resonance imaging (MRI). Moreover, diagnostic tools such as FNA biopsy are appropriate [21]. US imaging can be used as a first line method since it is available, does not include ionization radiation and can be combined with guided FNA and biopsy, as used in this case. Limited depth of penetration is one of the disadvantages of US, which can be overcome using CT. Among the advantages of CT are good anatomic localization and the less than 5 min taken to complete the neck scan. Its main disadvantages are the use of ionizing radiation, the fact that it requires intravenous iodinated contrast for optimum visualization of the neckanatomy, and possible dental artifacts, compromising image quality as well as limited soft tissue characterization. To overcome the use of ionizing radiation and limited soft tissue characterization, the surgeon can refer his patient to an MRI scan. MRI is contraindicated in certain patients, most commonly because of the insertion of metals and pacemakers, and is hardly tolerated by claustrophobic patients. However, its major advantage of improved soft tissue resolution compared to CT is found to be more suitable for the mass presented in this case [22].

Use of imaging modalities aid the surgeon to localize and make a first differential diagnosis although definitive diagnosis is achieved by histopathology and can differ from clinical diagnosis, which occurred in the case presented. Following the histopathology diagnosis, wide local excision with free margins is the suitable treatment for infiltrating angiolipoma. Surgical excision is curative for noninfiltrating lesions, as the recurrence rate is very low if excision is adequate. However, infiltrating angiolipomas have up to a 50% rate of recurrence [21]. Careful dissection must be achieved to avoid damage to the surrounding structures, such as the transverse cervical vessels as in this case. Moreover, the surgeon should bear in mind that hemostasis is crucial due to the blood vessels inside and surrounding the lesion. The overall prognosis for angiolipoma is good, as no malignant potential transformation has been reported [20,21]. However, these benign tumors do not spontaneously regress and can become larger, tenderer, and more cosmetically disfiguring, as in this case [20].

## 4. Conclusions

Using imaging modalities, such as US, FNA, contrast-enhanced CT and MRI, aids the surgeon to localize and make a first differential diagnosis of neck masses. Careful dissection must be achieved to avoid damage to surrounding structures and achieve proper hemostasis. Surgical excision with free margins is the “Gold standard” treatment and definitive diagnosis is achieved by histopathology.

## References

[1] Shah V.S., Harish M., Patel J.R., Shah N. (2013). Infiltrating angiolipoma of the cheek. BMJ Case Rep..

[2] Shahi A.K., Ash H., Chatterji K., Singh R. (2014). Cellular infiltrative angiolipoma of cheek in an infant. Natl. J. Maxillofac. Surg..

[3] Campos G.M., Grandini S.A., Lopes R.A. (1980). Angiolipoma of the cheek. Int. J. Oral Surg..

[4] Aniceto G.S., Saez R.S., Peńin A.G. (1990). Angiolipoma of the cheek: Report of a case. J. Oral Maxillofac. Surg..

[5] Ali M.H., el-Zuebi F. (1996). Angiolipoma of the cheek: Report of a case. J. Oral Maxillofac. Surg..

[6] Brahney C.P., Aria A.A., Koval M.H., Najjar T.A. (1981). Angiolipoma of the tongue: Report of case and review of literature. J. Oral Surg..

[7] Flaggert J.J., Heldt L.V., Keaton W.M. (1986). Angiolipoma of the palate. Report of a case. Oral Surg. Oral Med. Oral Pathol..

[8] Reilly J.S., Kelly D.R., Royal S.A. (1988). Angiolipoma of the parotid: Case report and review. Laryngoscope.

[9] Hamdan A.L., Mahfoud L., Rifai H., Rameh C., Fuleihan N. (2011). Infiltrative angiolipoma of the neck. Middle East J. Anesthesiol..

[10] Mesollela M., Di Martino M., Laguardia M., Galera F., Galli V. (2007). Angiolipoma of the larynx. Otolaryngol. Head Neck Surg..

[11] Furlong M.A., Fanburg-Smith J.C., Childers E.L.B. (2004). Lipoma of the oral and maxillofacial region: Site and sub classification of 125 cases. Oral Surg. Oral Med. Oral Pathol. Oral Radiol. Endodontol..

[12] Búa J.A., Luáces R., Franco F.L., García-Rozado A., Escudero J.C., Capdevila E.F., López-Cedruún J.L. (2010). Angiolipoma in head and neck: Report of two cases and review of the literature. Int. J. Oral Maxillofac. Surg..

[13] Hoeft S., Luettges J., Werner J.A. (2000). Infiltrating angiolipoma of the M. temporalis. Auris Nasus Larynx.

[14] Saydam L., Bozkurt M.K., Ugur M.B., Ozcelik T., Kutluay L. (2005). Angiolipoma of the neck: A case report. Ear Nose Throat J..

[15] Auo H.J., Kang J.M. (2009). Infiltrating angiolipoma of the nasopharynx: Adjacent to an aberrant internal carotid artery. Auris Nasus Larynx.

[16] Lin J.J., Lin F. (1974). Two entities in angiolipoma. A study of 459 cases of lipoma with review of literature on infiltrating angiolipoma. Cancer.

[17] Gerard N., Schultz D.A. (2009). Angiolipoma of the upper lip: Report of a case. J. Oral Maxillofac. Surg..

[18] Nicholson B., Prendergast T., Myers E.M. (2012). Angiolipoma of the neck. Am. Surg..

[19] Altug H.A., Sahin S., Sencimen M., Dogan N., Erdogan Ö. (2009). Non-infiltrating angiolipoma of the cheek: A case report and review of the literature. J. Oral Sci..

[20] Alvi A., Garner C., Thomas W. (1998). Angiolipoma of the head and neck. J. Otolaryngol..

[21] Yanase S., Nomura J., Matsumura Y., Kato H., Takeoka T., Imura H., Matsuura R., Nakanishi K., Tagawa T. (2011). Angiolipoma of the cheek: A case report with a literature review. Asian J. Oral Maxillofac. Surg..

[22] Blythe J., Pearce O.J., Tilley E.A., Brennan P.A. (2015). Contemporary Use of Imaging Modalities in Neck Mass Evaluation. Atlas Oral Maxillofac. Surg. Clin. N. Am..

